# Hospitalization Free-Survival, Adverse Drug Reactions, and Retention in Care Outcomes of an Outpatient Treatment Model for Cryptococcal Meningitis in PLWH in Maputo, Mozambique

**DOI:** 10.3390/tropicalmed11020048

**Published:** 2026-02-10

**Authors:** Maria Ruano Camps, Aleny Couto, Irénio Gaspar, Eudoxia Filipe, Idilia Nhamtumbo, Luis Armando, Gil Muvale, Ana Gabriela Gutierrez Zamudio, Rosa Bene, Jeff Lane, Florindo Mudender, Edy Nacarapa

**Affiliations:** 1 I-TECH Mozambique “International Training & Education Center for Health”, Bairro Sommerschield, Avenue Cahora Bassa N# 106, Maputo P.O. Box 1102, Mozambique; mariar@itech-mozambique.org (M.R.C.); idilian@itech-mozambique.org (I.N.); larmando@itech-mozambique.org (L.A.); rbene@itech-mozambique.org (R.B.); florindom@itech-mozambique.org (F.M.); 2 National STI/HIV/AIDS Program, Ministry of Health, Maputo P.O. Box 264, Mozambique; coutoaleny@gmail.com (A.C.); ireniogaspar@gmail.com (I.G.); filipeeudoxia@gmail.com (E.F.); 3 Centro de Referência de Alto-Maé “CRAM”, Maputo City Health Service, MoH, Maputo P.O. Box 264, Mozambique; gilmuvale@gmail.com; 4 Médecins Sans Frontières, Maputo P.O. Box 1224, Mozambique; gabriela.gz@me.com; 5 Department of Global Health, University of Washington, 908 Jefferson St., 12th Floor, Seattle, WA 98195, USA; lanej3@uw.edu

**Keywords:** cryptococcal meningitis, advanced HIV disease, hospitalization-free survival, ambulatory therapy model, Mozambique

## Abstract

Background: Cryptococcal meningitis (CM) remains a leading cause of mortality among people with advanced HIV disease (AHD) in sub-Saharan Africa. Current guidelines recommend induction therapy with amphotericin B and flucytosine, typically administered in an inpatient setting due to concerns over severe clinical presentation and drug-related toxicities. This requirement poses a significant burden on resource-limited health systems. We evaluated the real-world outcomes of a fully outpatient model for CM therapy in Maputo, Mozambique. Methods: A longitudinal retrospective cohort study was conducted at the Centro de Referência de Alto-Maé (CRAM), a specialized AHD outpatient clinic. We included 83 PLWH with laboratory-confirmed CM treated between October 2020 and December 2024. The primary outcome was hospitalization-free survival (HFS) within the first 10 weeks of treatment. Secondary outcomes included the frequency and severity of adverse drug reactions (ADRs), analysed by tracking haemoglobin (Hgb), potassium (K+), and creatinine (Creat) levels on days 1, 3, and 7 of induction therapy, and retention in care (RIC) at 6, 12, and 24 months. Statistical analyses included Kaplan–Meier survival estimates and paired t-tests. Results: The median age was 37 years (IQR: 27–42), 63.9% were male, and the median CD4 count was 62 cells/µL (IQR: 27–105). Most patients (95.2%) were symptomatic at presentation, and 56.6% had concurrent tuberculosis. For the 52 patients who completed the full induction protocol at CRAM, the HFS rate at 10 weeks was 84.6% (44/52), with an overall survival of 90.4% (47/52). ADR analysis (n = 52) showed a predictable pattern of mild, manageable toxicity: a significant decline in Hgb (11.2 ± 1.8 to 10.6 ± 2.0 g/dL, *p* < 0.001) and K+ (4.27 ± 0.66 to 3.86 ± 0.78 mmol/L, *p* = 0.008), and a transient increase in Creat (0.83 ± 0.42 to 1.13 ± 0.64 mg/dL, *p* = 0.001) from day 1 to day 3, with stabilization or a trend toward recovery by day 7. No significant differences in ADRs were found between single-dose (47%) and multiple-dose (53%) L-AmB regimens. RIC for the entire cohort (n = 83) was high at 81.9% at 6 months, declining to 74.0% at 12 months and 70.4% at 24 months. Conclusions: An ambulatory model for CM therapy is feasible and effective in a resource-limited setting, demonstrating high hospitalization-free survival, manageable and reversible adverse drug reactions, and excellent medium-term retention in care. These findings suggest potential benefits and provide support for re-evaluating the standard of inpatient care. They indicate that integrating outpatient CM management into advanced HIV disease (AHD) care packages could help alleviate health system burdens and may contribute to improved patient outcomes.

## 1. Introduction

Cryptococcal meningitis (CM) is a major concern and leading cause of meningitis in adults in Sub-Saharan Africa (SSA) and other regions with high prevalence of the Human Immunodeficiency Virus (HIV) infection, accounting for more than 100,000 incident cases of meningitis per year in the region. Globally, about 15% of HIV-related deaths are attributable to CM and 75% of these occur in SSA [1,2,3].

To date, studies on the management of CM advocate the administration of liposomal amphotericin B (L-AmB) during the induction phase which can be done in multiple doses or in a higher single dose, associated with oral flucytosine [4,5]. The induction phase has always been carried out in-hospital. This is due to the perception that CM involves patients in a serious clinical situation, as well as to ensure better management of medical complications and adverse reactions to medications [4].

Outpatient management of CM has not been recommended in existing national or international guidelines. However, noteworthy experience has been described by Medical Action Myanmar (MAM), a medical aid organization in Myanmar [6]. These instances demonstrated the potential to address limitations encountered in providing CM care in resource-limited settings. A retrospective cohort from Myanmar, including 66 patients, reported that using intravenous amphotericin B-deoxycholate (0.7–1.0 mg/kg) and oral fluconazole (800 mg orally/day), on an outpatient basis during the induction phase, saved 1029 days of hospital beds and had better survival outcomes [6]. In this context, additional evidence is required to show feasibility of a differentiated model of care that simplifies provision of CM care.

Until now, there has been a paucity of data describing the results of a completely ambulatory model of CM treatment. This analysis aimed to evaluate the results of an ambulatory model of CM management in the first 10 weeks of treatment at CRAM, Mozambique, between 2020–2024. It also describes the occurrence of adverse drugs reactions (ADR) to CM treatment in the sub-cohort of patients that received complete CM treatment at CRAM. Finally, it presents cohort retention data at 6 months.

## 2. Materials and Methods

### 2.1. Setting

The prevalence of HIV in Maputo City stands at 16.2% among individuals aged 15 and older [7], and a cumulative total of 170,161 people living with HIV (PLWH) were receiving antiretroviral therapy (ART) by mid-2024 [8].

Since 2013 MSF and I-TECH have managed the Centro de Referência do Alto-Maé (CRAM) in partnership with the Ministry of Health (MoH) as an outpatient clinic to offer ART treatment for HIV patients in Mozambique [7]. CRAM provides a range of outpatient services, with special focus on rapid and comprehensive screening and management of AHD [9]. As of the end of 2024, its cumulative registry included approximately 25,600 HIV-positive individuals, of whom 1700 remained actively engaged in care [10].

### 2.2. Study Design

This evaluation employed a longitudinal retrospective design to assess real-world implementation of CM management, with the aim of informing future program enhancements. The program implemented a longitudinal tracking system to evaluate patient outcomes by comparing initial baseline measurements with follow-up data. The included data were retrospectively collected as part of the screening and management of the AHD program from CRAM between October 2020 and December 2024.

#### 2.2.1. Eligible Participants

The eligibility criteria consisted of PLWH of all ages who had AHD and a CM diagnosis, and who were followed at CRAM between October 2020 and December 2024.

#### 2.2.2. Sampling and Sample Size

The sample encompassed the entire population of PLWH who had AHD and tested positive for cryptococcal antigen in their cerebrospinal fluid (CSF), as recorded in the AHD registration book at CRAM, in accordance with the specified eligibility criteria.

#### 2.2.3. Program Data Collection

The study team extracted routine clinical data from paper-based patient files during the period under analysis, between 2020 and 2024. Exposure variables were categorized into five fields: (1) demographic profile: gender and age; (2) immunological and nutritional assessments: T-CD4 cell count (Cell/μL) and body mass index (BMI); (3) treatment specifics: site of initial antifungal therapy for CM (CRAM or other HF), dose of L-AmB (single or multiple), ART status on admission (pre-ART, on ART, ART interruption); (4) CM symptoms: headache, vomiting, and meningeal signs [neck stiffness, Kernig’s sign, Brudzinski’s sign], decreased visual acuity or blindness, diplopia, agitation or unusual behaviour, photophobia, hypoacusis, seizures, reduced level of consciousness; (5) concurrent opportunistic infections (OIs): tuberculosis (TB), oral candidiasis, wasting syndrome, chronic diarrhoea and Kaposi sarcoma (Table 1).

Data collected from eligible PLWH were anonymized to remove identifying details: each patient was given an alphanumeric code, and the anonymized data were included in the data sheet. Spreadsheet data were exported to SPSS for further data analysis.

### 2.3. Outcome Data and Statistical Analysis of Data

Statistical analysis was performed using the statistical software IBM^®^ Statistical Package for the Social Sciences (SPSS) version 25. Graphs, including Kaplan–Meier survival curves, were generated using SPSS and exported for figure preparation.

#### 2.3.1. Primary Outcome

The primary outcome was hospitalization-free survival (HFS) within the first 10 weeks of treatment/follow-up, among participants with CM.

First, we characterized the study population by calculating frequencies and proportions with 95% confidence intervals (CI) for categorical variables, and medians with interquartile ranges (IQR) for continuous data. We then employed Kaplan–Meier analysis to estimate HFS within the 10 weeks of treatment/follow-up.

#### 2.3.2. Secondary Outcomes

Trends in haemoglobin (Hgb [g/dL), potassium (K^+^ [mmol/L]), and creatinine (creat [mg/dL]) levels on the first, third, and seventh day of CM treatment were assessed to identify related ADR.

We first assessed normality using the Shapiro–Wilk test, with results presented as mean ± standard deviation (SD). The dependent variables analysed were Hgb, K^+^, and creat. A paired *t*-test was used to compare values between the first, third, and seventh days of CM treatment, with a *p*-value < 0.05 considered statistically significant.

An additional secondary outcome, retention in care at 6, 12, and 24 months, was evaluated using Kaplan–Meier analysis to determine its probability over time.

#### 2.3.3. Definition of Hospitalization, HFS, and ART Experienced

• Hospitalization referrals were guided by two main criteria: first, the presence of clinical danger signs, including an altered level of consciousness (observed or reported), seizures, or other critical symptoms such as respiratory distress; and second, anticipated logistical barriers that would prevent regular clinic attendance, for instance, living a significant distance from the health facility without access to reliable transportation.
• HFS was defined as the proportion of patients who survived for at least 10 weeks following cryptococcal meningitis diagnosis without requiring inpatient admission at any point during this follow-up period.
• ART experienced was defined as an exposure of at least 30 days before CM diagnosis.

## 3. Results

### 3.1. Baseline Clinical and Demographic Characteristics of Patients with CM Followed Up at CRAM

Among the 83 CM patients, 38 (45.8%) received their first CM treatment at CRAM, and the remaining 45 patients (54.2%) had received at least one antifungal drug before CRAM admission.

**Figure 1:**
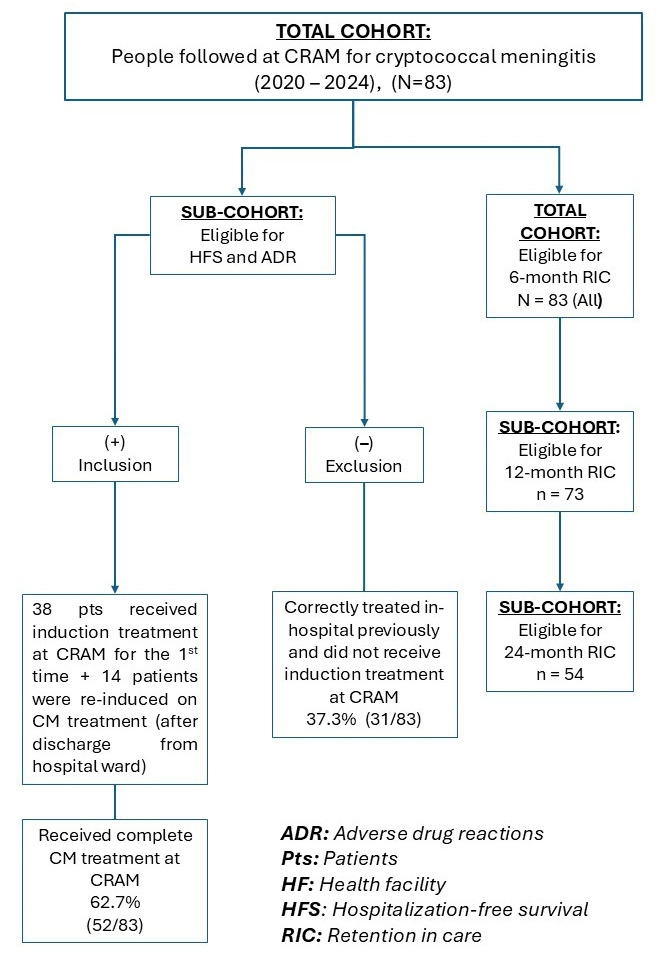
Flowchart of sub-cohort analysis. HF: Health facility, HFS: Hospitalization-free survival, CM: Cryptococcal meningitis, CRAM: Centro de Referência Alto-Maé, Pts: patients.

Of those 45 patients previously treated, 14 (31.1%) were reinitiated on antifungal therapy, due to inconsistent/incomplete previous treatment (generally without amphotericin B and/or flucytosine). The remaining 31 patients were considered correctly treated prior to arrival at CRAM and follow up continued without the need of induction therapy. The full cohort (n = 83) was included in the retention analyses, while the sub-cohort of patients who completed treatment for CM (n = 52) was used in the analyses of hospitalization-free survival and treatment-related adverse events (see Figure 1).

Between October 2020 and December 2024, 83 individuals with CM were enrolled at CRAM.

Median age at admission was 37 years (interquartile range [IQR]: 27–42), with 36 (43.4%) falling within the 35–44 age range. Furthermore, 53 (63.9%) of the participants were male (Table 1).

Among the 83 patients with CM, the median CD4 cell count was 61 cells/µL (IQR: 27–105). Notably, 34 (41.0%) had a CD4 cell count below 50 cells/µL, while 39 (50.0%) had a body mass index (BMI) under 18.5 kg/m^2^, indicating malnutrition. Forty-four patients (53.0%) were ART experienced according to our definition and 39 (47.0%) were considered naïve to ART (Table 1).

Seventy-nine patients (95.2%) were symptomatic. The most common signs and symptoms included headache (78, 94%), vomiting (27, 32.5%), and meningeal signs (26, 31.3%). Other reported symptoms were decreased visual acuity (19, 22.9%), agitation or unusual behaviour (16, 19.3%), photophobia (16, 19.3%), hypoacusis (8, 9.6%), diplopia (8, 9.6%), seizures (6, 7.2%), and coma (1, 1.2%) (Table 1).

Of 79 patients, 34 (43.0%) had one lumbar puncture (LP), and 45 (57.0%) had multiple—38 (48.1%) with two to five and seven (8.9%) with ≥six. In four cases (5.1%) LP was not successful. Opening cerebrospinal fluid pressure (OP CSF) was recorded in 70 cases: nine (12.9%) were normal (≤20 cmH_2_O), 29 (41.4%) moderately elevated (21–40 cmH_2_O), and 32 (45.7%) severely elevated (≥41 cmH_2_O).

Concurrent OIs were also common: 47 patients (56.6%) had a concomitant TB diagnosis, 18 (21.7%) presented with oral candidiasis, and 15 (18.1%) exhibited wasting syndrome. Other conditions included chronic diarrhoea (6, 7.2%) and Kaposi sarcoma (5, 6%) (Table 1).

### 3.2. HFS Within the First 10 Weeks of Treatment Among Patients with CM

**Figure 2:**
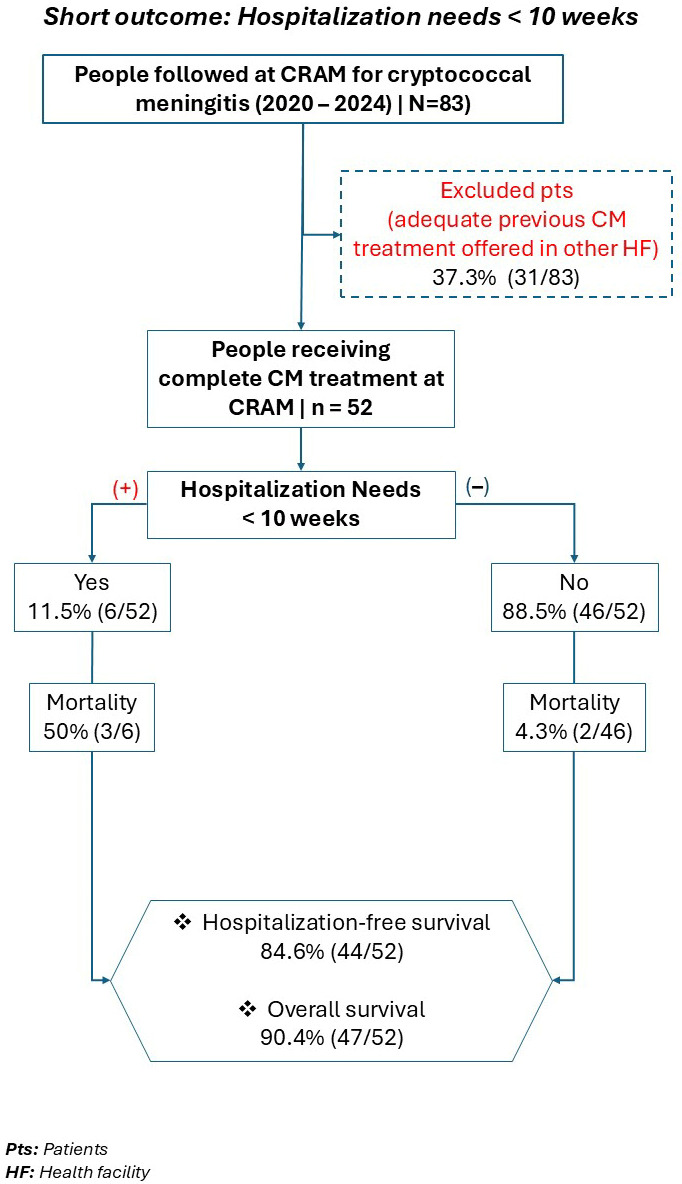
Short outcome: Hospitalization needs < 10 weeks. HF: Health facility, HFS: Hospitalization-free survival, CM: Cryptococcal meningitis.

Regarding HFS analysis, we included 52 patients who completed the full CM treatment protocol at CRAM. The remaining 31 patients were excluded from the analysis, as they were presumed to have been appropriately induced on CM treatment prior to their arrival at CRAM, in a hospital setting under inpatient care. Among those 52 patients, six (11.5%) required hospitalization during the first 10 weeks of treatment. Mortality among these patients requiring hospitalization was 50% (three out of six died). Two patients died at home, 2 and 23 days after initiation of treatment, respectively. HFS reached 84.6% by week 10 of treatment. Overall survival was 90.4% (Figure 2).

**Figure 3:**
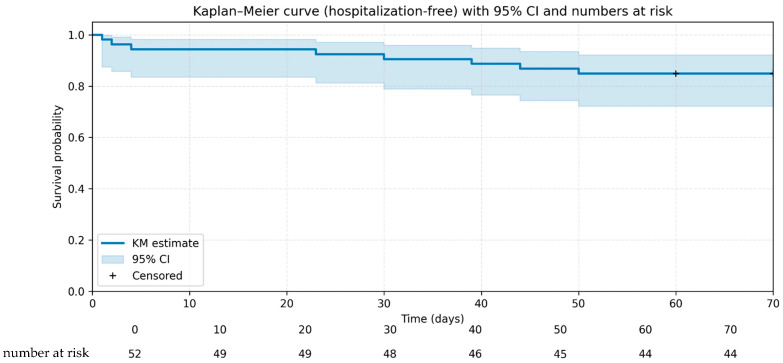
Kaplan–Meier plot estimating the probability of HFS within the first 10 weeks of outpatient antifungal therapy for CM.

Kaplan–Meier analysis indicates a high probability of HFS within the first 10 weeks of treatment follow-up, with an estimated incidence of 84.6% at week 10 (Figure 3).

When analysing overall mortality in the cohort of patients who completed treatment for CM at CRAM, we observed five deaths during the first 10 weeks of follow-up. The overall survival rate of the cohort was 90.4% (47/52).

### 3.3. ADR of Treatment (Frequency and Severity) Among Patients with CM

Regarding analysis of ADR of CM treatment, we considered the sub-cohort of patients that received complete CM treatment at CRAM (n = 52). The remaining 31 patients were excluded, as they did not receive L-AmB at CRAM and no accurate laboratory data was available.

The analysis of laboratory parameters revealed a clear pattern of initial minimum toxicity followed by stabilization, in patients receiving L-AmB and flucytosine induction therapy. Of the 52 patients who completed treatment for CM at CRAM, 27 (52%) received the 7-day protocol with intravenous liposomal L-AmB. The following 25 patients (48%) received the single-dose L-AmB protocol, introduced at CRAM in September 2022. No differences were observed in the occurrence of adverse effects between the two protocols; therefore, toxicity data are presented jointly.

Haemoglobin (Hgb) levels showed a mild decline of 0.6 g/dL, from a baseline mean (plus ± SD) of 11.2 ± 1.8 g/dL on Day 1 to 10.6 ± 2.0 g/dL by Day 3 (*p* < 0.001), suggesting the early onset of drug-induced anemia. No significant recovery was observed, with levels remaining at 10.6 ± 1.8 g/dL through Day 7 (*p* = 0.738).

Potassium (K^+^) levels, which were within the normal range at 4.27 ± 0.66 mmol/L on Day 1, showed a mild decline of 0.26 mmol/L to a below-normal nadir of 3.86 ± 0.78 mmol/L by Day 3 (*p* = 0.008), confirming hypokalaemia. By Day 7, a trend toward recovery was observed, with levels rising to 3.97 ± 0.61 mmol/L (*p* = 0.175) (Table 2).

Creatinine (Creat), showed a mild increase of 0.39 mg/dL, rising from a normal baseline of 0.83 ± 0.42 mg/dL on Day 1 to 1.13 ± 0.64 mg/dL on Day 3 (*p* = 0.001), indicating mild nephrotoxicity. Encouragingly, by Day 7, the mean decreased to 0.99 ± 0.43 mg/dL (*p* = 0.370), suggesting a trend toward recovery (Table 2).

### 3.4. Retention in Care (RIC) Among Patients with CM (At 6, 12, and 24 Months)

At the time of analysis, 83 patients with CM had completed 6 months of follow-up, 73 had completed 12 months, and 54 had reached 24 months of clinical follow-up.

**Figure 4:**
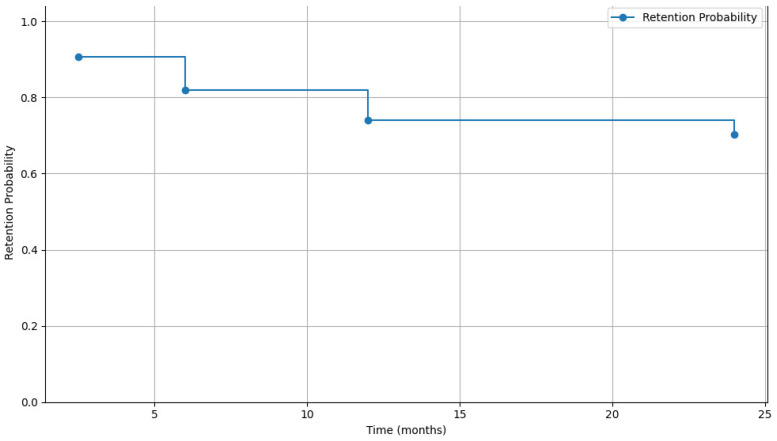
Retention Trends in CM Follow-up.

As expected, the analysis revealed a decline in patients RIC over time, dropping from 81.9% at 6 months to 70.4% at 24 months (*p* < 0.001) (see Figure 4).

Death was the leading cause of loss of follow-up, accounting for 60% of losses at 6 months, 52% at 12 months, and 43% at 24 months of follow-up. The remaining losses of follow-up were evenly distributed between cases of discontinuation and patient-requested transfers to other healthcare facilities (see Table 3).

## 4. Discussion

### 4.1. High HFS and Overall Survival

This study demonstrates that an ambulatory model for CM therapy is feasible and effective, in a resource-limited setting. Our findings demonstrate favourable clinical outcomes, with high rates of hospitalization-free survival and overall survival among patients who received ambulatory treatment for CM. Specifically, 84.6% (44/52) remained alive without requiring hospitalization during the critical first 10 weeks of therapy, while overall survival was 90.4% (47/52), including those referred for inpatient care post-diagnosis who ultimately survived.

When compared to previous data from the region, these results suggest a substantial improvement in prognosis. A study conducted in Maputo and published in 2024 reported a 12-week mortality rate of 37.9% [11], whereas our cohort achieved an overall survival of 90.4% during the first 10 weeks of treatment. This contrast underscores significant advances in CM management, reflecting an approximate fourfold reduction in early mortality.

Additional multicentre data reinforce the relevance of these findings. A prospective cohort study conducted in sub-Saharan Africa (SSA) which aimed to describe short-term mortality outcomes of HIV-associated CM, found that 10-week all-cause mortality was similar between antiretroviral therapy (ART)-experienced and ART-naïve patients—38% versus 36%, respectively (*p* = 0.64; hazard ratio [HR], 1.03; 95% CI: 0.80–1.32; *p* = 0.82) [12].

The ten-week survival rate observed in the CRAM cohort also exceeded that of other studies conducted in Southern Africa. For instance, compared to the Ambition study cohort, which reported a 10-week survival of 75.2% in the L-AmB group, CRAM achieved a rate 15 percentage points higher over the same period among patients who received ambulatory treatment for CM [13].

Similarly, a prospective cohort study conducted in Uganda between 2022 and 2023 evaluated adult CM patients treated with the single-dose L-AmB regimen. Among 179 participants, the 10-week survival probability estimated via the Kaplan–Meier method was 68.6% (95% CI: 61.6–76.3%). While this represents one of the strongest survival outcomes reported outside a controlled trial setting, approximately one-third of patients still died within 10 weeks, underscoring the persistent burden of CM despite improved therapy [14].

Taken together, these comparisons highlight the relative success of the CRAM model. We believe a key factor contributing to the improved survival is the delivery of treatment at a primary-level health facility, within a setting where systematic screening for cryptococcal antigenemia is being more comprehensively implemented, which enables earlier diagnosis and intervention. This approach, combined with same-day clinical response, likely facilitated timely initiation of therapy and led to better outcomes. An additional factor that may have contributed to the higher survival rate was the provision of home-based care by family members, under the guidance of the clinical team. In hospital wards within resource-limited settings, such as in Mozambique, these forms of care are not always available, which may lead to an increased risk of morbidity and mortality.

Our study confirms that outpatient CM induction therapy is feasible and highly effective in resource-limited settings. The 84.6% HFS rate over the initial 10 weeks provides compelling real-world evidence that challenges the traditional reliance on prolonged inpatient care. These findings align with and reinforce limited existing literature on ambulatory CM management [6].

While a direct comparison with hospitalized patients from the same setting was not feasible in this study. Future studies should include a concurrent hospitalized control group to further validate the advantages of the outpatient model.

### 4.2. Manageable and Reversible ADRs

Initial safety concerns regarding outpatient CM management were rapidly mitigated, given the excellent safety outcomes and minimal serious adverse drug reactions (ADRs). Declines in haemoglobin and serum potassium were limited, and no hospitalizations occurred due to laboratory changes. Serum creatinine increased mildly but returned to baseline within the first week.

L-AmB showed a favourable safety profile under both the 10 mg/kg single-dose and 3 mg/kg/day 7-day regimens. This was supported by baseline renal and electrolyte monitoring and intravenous potassium supplementation for patients with normal renal function; only six patients required extra oral supplementation due to hypokalaemia.

ADR analysis revealed a predictable pattern of mild, manageable toxicity. We observed a statistically significant but clinically modest decline in haemoglobin and potassium, and a transient increase in creatinine within the first three days, with stabilization or recovery by day seven. These findings align with the known toxicity profile of L-AmB and confirm that outpatient monitoring is feasible without hospitalization. The absence of significant differences between regimens further supports the flexibility of this approach.

### 4.3. High RIC at 6 Months

Most studies on CM have concentrated on short term mortality, typically assessed within 10 weeks of diagnosis, whereas medium term retention has received comparatively limited attention. Among 83 CM patients assessed, 52 of whom initiated treatment at CRAM and 31 of whom were referred post-induction from other facilities, the retention remained high: 81.9% (68/83) at six months, 74% (54/73) at 12 months, and 70.4% (38/54) at 24 months. The retention outcomes in the CRAM cohort can be attributed to the fact that patients receive comprehensive care in a single facility, eliminating the need to navigate multiple health services.

By incorporating induction therapy within a specialized ambulatory clinic, the CRAM model effectively bridges the gap between inpatient and outpatient care. This integration mitigates patient drop-off commonly driven by the complexity of care pathways for advanced HIV disease (AHD), which frequently leads to disengagement [15]. CRAM’s “one-stop-shop” approach, encompassing cryptococcal meningitis (CM) treatment, antiretroviral therapy (ART), and management of other opportunistic infections (OIs) addresses structural barriers and care fragmentation, thereby promoting sustained patient engagement. These findings suggest that a dedicated outpatient model for AHD is not only feasible but may outperform traditional systems in retaining patients over time.

### 4.4. Other Findings: High Burden of Concurrent TB

In our cohort, TB was diagnosed and treated in 56.6% of patients (n = 47), reflecting the CRAM’s rigorous and systematic TB screening protocol at entry. This protocol includes clinical examination, digital chest radiography (CXR), TB LAM and other molecular tests as indicated, as well as focused assessments using sonography and retinoscopy to detect extrapulmonary TB in patients with CD4 counts below 100 cells/μL.

A study on short-term mortality in 678 CM patients in four African countries (Malawi, Tanzania, Zambia, and Cameroon), recruited between 2013 and 2016, reported TB co-infection in 98 patients (14.5% of CM enrolled patients) [12].

The remarkably high rate observed in our Mozambican cohort underscores the critical importance of integrated “AHD care packages” that bundle CrAg screening with intensive TB diagnostics, such as systematic symptom screening and WHO-recommended urine lateral flow lipoarabinomannan (LF-LAM) testing. The presence of TB in over half of patients highlights a key challenge in CM management: TB co-infection can complicate clinical presentation, exacerbate drug interactions and toxicities, and potentially worsen outcomes. Therefore, the success of the CRAM ambulatory model may not solely be due to the effective delivery of antifungal therapy but could also stem from its capacity to provide simultaneous, coordinated management for TB and other AHD-defining conditions within a single, patient-centred facility. This integrated approach is essential for addressing the complex syndemic of HIV, TB, and cryptococcosis in the region.

### 4.5. Strengths and Limitations

This study has several strengths. Firstly, it provides critical real-world evidence from a programmatic setting in SSA, demonstrating the feasibility and effectiveness of a fully ambulatory model for CM induction therapy, a care approach not yet widely endorsed in international guidelines. The cohort represents a typical, highly vulnerable population with AHD, characterized by severe immunosuppression and a high burden of comorbidities like TB, thus enhancing the generalizability of our findings to similar resource-limited contexts. Furthermore, the analysis of retention over a 24-month period provides valuable long-term insights that go beyond the typical short-term outcomes reported in many CM studies.

However, several limitations must be acknowledged. The single-centre, observational design of our study limits causal inference and may affect the generalizability of findings to other settings with different healthcare infrastructures or patient populations. The relatively small sample size reduces analytical power and the ability to detect rare adverse events. The retrospective collection of data from routine clinical records carries a risk of missing or inconsistently recorded information, such as the precise grading of ADR or detailed neurological outcomes. The exclusion of 31 patients who received induction therapy elsewhere, while methodologically sound for the HFS analysis, means our primary safety and efficacy results are based on a subset of the total cohort managed at the clinic. Future prospective, multicentre studies with larger sample sizes are warranted to confirm these promising findings. Finally, it could be argued that the exclusion of patients previously treated under inpatient care might introduce a selection bias, resulting in a cohort composed of individuals with greater clinical stability or lower disease severity. However, the HFS sub-cohort included 14 cases of patients who had been hospitalized in other health facilities and required retreatment from the outset. For this reason, we consider that such bias was effectively mitigated.

## 5. Conclusions

Despite the widespread perception that CM is a severe condition that can only be managed in a hospital setting under inpatient care, the results from the CRAM cohort demonstrate that this disease can be effectively treated and achieve excellent outcomes without the need for hospitalization.

It is likely that CRAM’s status as a primary-level health unit has facilitated earlier access to care and diagnosis of advanced HIV disease and cryptococcal infection, which may have contributed to improved treatment outcomes.

## Figures and Tables

**Table 1:** Demographic and clinical characteristics of 83 people followed at CRAM and diagnosed/treated for CM, 2020–2024. yrs: years.

		Column, N (%)	95% CI
		N = 83	
Gender	Men	53 (63.9)	(53.2–73.6)
Age	Median, interquartile range (IQR)	37	27–42
Age band	≤24 yrs old	11 (13.3)	(5.8–22.5)
	25–34 yrs old	21 (25.3)	(16.5–36.0)
	35–44 yrs old	36 (43.4)	(32.5–54.8)
	45–54 yrs old	11 (13.3)	(6.8–22.5)
	≥55 yrs old	4 (4.8)	(1.3–11.9)
Body mass index band (Kg/m^2^)	BMI < 18.4	39 (50.0)	(39.1–60.9)
CD4 cell count cells/µL	Median, interquartile range (IQR)	61	27–105
CD4 band at CRAM entry	CD4 < 50 Cell/µL	34 (41.0)	(30.3–52.3)
	CD4 [51–99] Cell/µL	27 (32.5)	(22.7–43.6)
	CD4 ≥ 100 Cell/µL	22 (26.5)	(17.4–37.4)
Antifungal therapy prior to CRAM	Antifungal therapy initiated at CRAM	38 (45.8)	(35.4–56.5)
	Antifungal therapy prior to CRAM	45 (54.2)	(43.5–64.6)
Induction therapy at CRAM	No (amphotericin received at other HF)	31 (37.3)	(27.5–48.0)
	Yes (amphotericin received at CRAM)	52 (62.7)	(52.0–72.5)
Amphotericin	Single dose	39 (47.0)	(36.5–57.7)
	Multiple dose	44 (53.0)	(42.3–63.5)
ART vs. CM timeline	Pre-ART or ≤30 days on ART	39 (47.0)	(36.5–57.7)
	Experienced ART > 30 days and active	21 (25.3)	(16.9–35.4)
	Experienced ART > 30 days but with ART interruption on admission	23 (27.7)	(19.0–38.0)
Clinical features:	Symptomatic	79 (95.2)	(88.9–98.4)
	Headache	78 (94.0)	(87.3–97.7)
	Vomiting	27 (32.5)	(23.2–43.1)
	Agitation/bizarre behavior, incoherent speech, delusions and/or hallucination	16 (19.3)	(11.9–28.7)
	Meningeal signs	26 (31.3)	(22.1–41.8)
	Decreased visual acuity/blindness;	19 (22.9)	(14.9–32.8)
	Photophobia	16 (19.3)	(11.9–28.7)
	Diplopia/CN-VI palsy	8 (9.6)	(4.7–17.4)
	Hypoacusis	8 (9.6)	(4.7–17.4)
	Seizures	6 (7.2)	(3.1–14.3)
	Reduced GCS	1 (1.2)	(0.1–5.5)
Frequency of lumbar punctures (LP)	Not succeeded	4 (5.1)	(1.4–12.5)
	1 LP (initial)	34 (43.0)	(32.1–54.6)
	2–5 LP	38 (48.1)	(36.9–59.5)
	≥6 LP	7 (8.9)	(3.6–17.5)
Opening cerebrospinal fluid pressure (OP CSF)	OP CSF ≤ 20 cmH_2_O	9 (12.9)	(6.1–23.0)
	OP CSF [21–40] cmH_2_O	29 (41.4)	(29.9–53.7)
	OP CSF ≥ 41 cmH_2_O	32 (45.7)	(34.0–57.9)
Other active opportunistic disease:	Active TB	47 (56.6)	(45.9–66.9)
	Active oral candida	18 (21.7)	(13.9–31.4)
	Active chronic diarrhea	6 (7.2)	(3.1–14.3)
	Active Kaposi sarcoma	5 (6.0)	(2.3–12.7)

IQR: interquartile range, GCS: Glasgow Coma Scale, CSF: Cerebrospinal fluid, OP: Opening pressure, LP: Lumbar puncture, CN: Cranial nerve.

**Table 2:** Adverse drug reactions of antifungal treatment for cryptococcal meningitis at CRAM, Mozambique 2020–2024, | (N = 52).

Parameter (Day 1 vs. Day 3)	1st Day (Mean ± SD), [n]	3rd Day (Mean ± SD), [n]	Mean Paired Diff	t-Value	*p*-Value	
**Hgb (g/dL)**	11.2 ± 1.8 (n = 52)	10.6 ± 2.0 (n = 38)	**−0.6**	+4.73	**<0.001**	**↓**
**K+ (mmol/L)**	4.27 ± 0.66 (n = 49)	3.86 ± 0.78 (n = 40)	**−0.26**	−2.81	**0.008**	**↓**
**Creat (mg/dL)**	0.83 ± 0.42 (n = 52)	1.13 ± 0.64 (n = 40)	**+0.39**	+3.46	**0.001**	**↑**
**Parameter ** **(Day 3 vs. Day 7)**	**3rd Day (Mean ± SD), [n]**	**7th Day (Mean ± SD), [n]**	**Mean Paired Diff**	**t-value**	***p*** **-value**	
**Hgb (g/dL)**	10.6 ± 2.0 (n = 38)	10.6 ± 1.8 (n = 38)	**+0.05**	+0.35	0.738	---
**K+ (mmol/L)**	3.86 ± 0.78 (n = 40)	3.97 ± 0.61 (n = 38)	**+0.11**	+0.23	0.175	---
**Creat (mg/dL)**	1.13 ± 0.64 (n = 40)	0.99 ± 0.43 (n = 40)	**−0.14**	−0.91	0.370	---

SD = Standard Deviation; n = sample size; Diff = Differences; **↓** decline; **↑** rise.

**Table 3:** Retention and Attrition Trends in MCC Follow-up.

Follow-Up Period	Retained, N (%)	Censored = Died + Lost to Follow-Up (LFU) + Transferred Out (TO)				Total, N (%)	Cochran–Armitage Test for Trend, *p*-Value
		Censored, N (%)	Died, N (%)	LFU, N (%)	TO, N (%)		
6 months	68 (81.9)	15 (18.1)	9 (10.8)	3 (3.6)	3 (3.6)	83 (100)	<0.001
12 months	54 (74.0)	19 (26.0)	10 (13.7)	5 (6.8)	4 (5.5)	73 (100)	
24 months	38 (70.4)	16 (29.6)	7 (13.0)	4 (7.4)	5 (9.3)	54 (100)	

## Data Availability

The datasets utilized in this study are available from the corresponding author upon reasonable request; however, they are not publicly accessible due to privacy constraints.

## Abbreviations

The following abbreviations are used in this manuscript:

aOR	Adjusted odds ratios
ADR	Adverse drug reactions
AHD	Advanced HIV disease
ART	Antiretroviral therapy
CI	Confidence intervals
CM	Cryptococcal meningitis
CrAg	Cryptococcal antigen
CSF	Cerebrospinal fluid
CRAM	Centro de Referência de Alto-Maé
IQR	Interquartile range
HF	Health facility
HFS	Hospitalization-free survival
I-TECH	International training and education center for health
L-AmB	Liposomal amphotericin B
OIs	Opportunistic infections
PHC	Primary healthcare centre
PLWH	People living with HIV
MoH	Ministry of Health
MSF	Médecins Sans Frontières
SD	Standard deviation
SSA	sub-Saharan Africa
TB	Tuberculosis
